# Body, boundary, power: the impact of gender identity on professional relationships and working conditions in coaching

**DOI:** 10.3389/fpsyg.2026.1770576

**Published:** 2026-03-05

**Authors:** Nedim Malkoç, Hakan Sunay, Ümran Sarıkan, Ömer Ünal

**Affiliations:** 1 Department of Exercise and Sports Sciences, Hamidiye Faculty of Life Sciences, University of Health Sciences, Istanbul, Türkiye; 2 Department of Sports Management, Faculty of Sport Sciences, Ankara University, Ankara, Türkiye; 3 Department of Sports Management, Institute of Health Sciences, Ankara University, Ankara, Türkiye

**Keywords:** coaching, gender equality, gender roles, glass ceiling, invisible labor

## Abstract

**Background:**

The coaching profession plays a critical role in athlete development and building an egalitarian culture in sports. However, professional practice is often shaped by gender-based stereotypes, prejudices, and structural limitations. This study aims to examine the multilayered and systemic nature of gender inequality in the coaching profession.

**Methods:**

A qualitative research approach using a phenomenological design was employed. The experiences of active male and female coaches (*n* = *10*) were investigated through semi-structured in-depth interviews. Participants were selected using criterion sampling to ensure gender balance across male-dominated, female-dominated, and neutral sports branches. Data were analyzed using thematic analysis.

**Results:**

The analysis identified three main themes constraining coaches' professional lives: Invisible Labor, Glass Ceiling & Glass Cliff, and Sexual Objectification. Findings revealed that female coaches struggle with a “Glass Ceiling” where their competencies are questioned, career paths are restricted, and they bear emotional burdens imposed by gender norms. Conversely, male coaches are compelled to establish excessive distance and formality, particularly with female members, to avoid being perceived as potential objects of sexual threat. This creates a “Communication Barrier” that limits their professional effectiveness and market volume.

**Conclusions:**

Gender inequality in coaching possesses a dual structure, encompassing not only the disadvantaged position of women but also structural challenges for male coaches exposed to restrictive hegemonic masculinity norms. To achieve genuine and inclusive equality in sports, policies must be broadened to address the specific constraints faced by both genders.

Coaches play a pivotal role in fostering a positive youth and sport experience across physical, psychological and social dimensions (Merkel, 2013). A coach is not only responsible for formulating, planning, organizing, managing and supervising the training process, but also holistically influences athletes' physical, mental, intellectual, educational and social development (Rynne and Mallett, 2012; Smith, 2003). Coaches' knowledge, skills, competencies, and experiences are among the fundamental factors that determine the coaching activities to be put into practice in order to effectively manage the athlete's developmental process and build solid coach-athlete relationships (Koh and Tan, 2022; Gilbert and Côté, 2013).

The expansive roles held by coaches highlight that they are critical not only in athlete development, but also in building an egalitarian culture in sport. Indeed, sport is recognized as an important phenomenon for promoting and empowering gender equality (IOCOC, 2024). However, gender inequality in sport through stereotypes, biases and discrimination directly affects coaching practices and brings structural limitations to this profession. Research indicates that gender inequality in sport does not stem solely from coaching practice, but is also a product of a broader social context (Vaquero-Cristóbal et al., 2024). Therefore, coaches' responsibilities are not limited merely to enhancing sporting performance; they also need to emerge as role models who promote gender equality.

When gender inequality is considered through its many causes and consequences, it exerts a significant influence in the world of sport. On the other hand, particularly in coaching, the persistence of inconsistencies due to gender inequality appears to be linked to a variety of variables such as socio-cultural, organizational and interpersonal relationship-based barriers (Banwell et al., 2021; Dias et al., 2025). In this context it is stated that issues of gender inequality in sport and in the coaching profession are made up of a wide array of problems that cannot be reduced to a single cause.

Gender-based stereotypes are typically defined as sets of behaviors or attitudes attributed to male or female individuals, framed within a given social context, used to classify roles and often serve as the source of differences between men and women (Vaquero-Cristóbal et al., 2024; Herdt, 1996). On this ground, the concept of gender inequality is one of the biggest obstacles at the macro level to achieving full equality in sport (Meier, 2005). Indeed the 2030 Sustainable Development Agenda has prioritized promoting gender equality, removing gender stereotypes and preventing discrimination (United Nations, 2015). The International Olympic Committee (IOC) plays a critical role in promoting gender equality—a fundamental human right accepted as a basic principle of the Olympic Charter (IOCOC, 2024; Dias et al., 2025). Likewise, official documents such as the European Sport Coaching Policy provide key guidelines summarizing coaching roles, competencies and qualifications which may differ between sports and countries (Lara-Bercial et al., 2017; Bales and Moustakas, 2022).

At the community level, the Gender Equality Strategy 2020-2025 (European Commission) (European Commission, 2024). created a series of directives to strengthen balance between men and women in leadership positions within sport organizations, though the effectiveness of these policies remains debated. Meanwhile, it emphasizes the need for more research on gender equality and equity in various fields of sport. However these debates are not limited to institutional regulations and legal policies; gender inequality is a phenomenon that also needs to be addressed in its historical and ideological dimensions (Bekiari, 2023). In this regard, the struggles of women against centuries-old inequalities laid the groundwork for the birth of modern gender discourse and especially feminist thought.

In the eighteenth century, with the Enlightenment, women began to challenge political, cultural and social inequalities; especially in the nineteenth century, this struggle paved the way for the emergence of feminism (Taş, 2016). Feminism is regarded not merely as a social movement but also as an intellectual perspective that explains women's social position and criticizes male-dominated structures. In this context, the distinction between biological sex and social gender is critical. Biological sex is innate, whereas social gender is constructed on the roles, identities and expectations assigned to men and women. Ultimately, these categorical divisions between women and men lead to the formation of a patriarchal social order (Oakley, 1985; Hill, 2003; Scott, 2007; Berktay, 2009; Butler, 2010; Mermutlu and Taş Çetin, 2023; Mazerolle et al., 2015).

In its modern sense, feminism is associated with the women's movement and the effort to develop women's social role (Heywood, 2013). Feminism aims to understand women's rights in opposition to male dominance and is a movement style with sociological, political and moral dimensions. This movement questions the role of gender in individual and social identity formation. The chasm between being biologically male or female and socially male or female laid the groundwork for feminism (Eliuz, 2011; Atan, 2015). Radical feminism, in particular, links the origins of gender inequalities to the patriarchal order, emphasizing how inequality is reproduced in every area from family to education, from work life to religion with the slogan “The personal is political” (Mermutlu and Taş Çetin, 2023).

When this theoretical framework is applied to the field of sport, it is stated that sport has continued as a male-dominated area from past to present (Vaquero-Cristóbal et al., 2024; Díez-Mintegui, 2003). Historically, the socialization processes associated with sport, to which values such as success and prominence are attributed, have become highly androcentric through educational-sport environments that reproduce and legitimize gendered stereotypes from early ages. At precisely this point, feminism highlights the gender-based inequalities encountered by female coaches by criticizing the androcentric order and rendering visible the ways in which women are placed in secondary positions in sport. These inequalities are not only structural but also cultural, symbolic and practical, forming reflections of patriarchal and male-centered social order in the field of sport.

Traditionally, sport has been a sector dominated by men, and progress in gender equality within it is impeded by the presence of social structures which associate sport—particularly those involving attributes such as power, endurance and speed that occur in highly competitive and combative contexts—with masculinity (Fink, 2008; Amin et al., 2023). Attributes like strength and endurance are assigned to men, while activities involving aesthetics, concentration and flexibility are attributed to women and to the socio-cultural origin of modern sport (Plaza et al., 2017). This relationship also has a clear cultural bias dimension. Sport disciplines tend to be classified as “masculine” or “feminine” according to their dominant features. Moreover, in certain geographic regions, the fact that certain sports are practiced more by men or by women affects how both genders perceive these sports in terms of gender roles (Matteo, 1988). In this respect we see that sport is not a neutral field; due to cultural biases some branches may be coded as “men's sport” or “women's sport”. Consequently the perceptions of sport held by female and male athletes are not independent of these prejudices. Furthermore, these prejudices shape not only the attitudes of athletes toward sports branches, but also the attitudes of coaches. Ultimately the relationship between sport and coaching is a whole, and the forms of that relationship influence one another in one way or another.

Although the participation of individuals of different genders in the world of sport has increased in recent years, various challenges and discriminations persist. Especially in coaching positions there is unequal representation between genders (Barreira, 2021; Norman, 2012). For this reason, the participation of women in the coaching profession is increasingly a subject of interest in both scientific literature and sport organizations (Lavoi and Baeth, 2017).

At this point, understanding gender inequality in sport requires not only looking at micro-level interactions but also at structural factors at individual, institutional and cultural levels. Studies based on extensive literature review, in the framework of Urie Bronfenbrenner's ecological systems theory, have examined the factors influencing coaches' experiences at four fundamental levels: individual, interpersonal, organizational and socio-cultural (LaVoi and Dutove, 2012; Cardoso et al., 2022). The individual level represents the personal, biological and psychological characteristics (i.e., emotions, beliefs and personality) that may facilitate or hinder a coach's career progress. The second level, interpersonal relationships, represents the impact of colleagues, family and friends on their development. The third level, organizational, represents the sport policies, professional practices and opportunities (or lack thereof) for female coaches. The fourth and furthest level consists of socio-cultural factors representing norms and cultural systems that influence coaches' trajectories. Therefore, increasing the participation of female coaches in sport is a challenge characterized by a complex interaction between individual characteristics, organizational aspects and cultural systems (LaVoi and Dutove, 2012; Cardoso et al., 2022).

Investigating gender inequality associated with the concept of coaching in sport requires adopting an interdisciplinary approach to understand entrenched societal judgments and behaviors. In a phenomenon as complex as sport, understanding coaches' lived experiences regarding gender-based inequality in their professional lives is thought to help identify needs and tools to reduce gender-based differences in their coaching practice.

Thus, the aim of this study is to delve deeply into the multi-layered nature of gender-based inequalities in the coaching profession, examining them on individual, social and ethical levels. The purpose is not only to make the problem visible, but also to question the structural and social ground behind this issue and to invite a rethinking of prevailing understandings in sport. Furthermore, explaining the phenomenon of gender-based inequality also means questioning the societal stereotypes that prevent the sport coach from meaningfully constructing his or her own existence. To ensure conceptual clarity, this study distinguishes between gender equality, defined as the equal rights and opportunities for all coaches regardless of gender, and gender equity, which refers to the fairness of treatment through structural supports that address historical disadvantages. While ‘inequality' describes the current problematic state, ‘equity' serves as the necessary mechanism to achieve sustainable ‘equality' in the coaching profession.

In this study, the analysis is primarily informed by a liberal feminist framework combined with critical feminist perspectives. While liberal feminism helps address institutional barriers and the quest for equal representation (e.g., Glass Ceiling), a critical feminist lens allows for a deeper exploration of how power dynamics and hegemonic masculinity shape the everyday lived experiences and ‘invisible labor' of both female and male coaches.

## Method

The design of this study was determined as phenomenology, one of the qualitative research approaches. Phenomenology aims to focus on phenomena experienced by participants in their daily lives, generally known but not fully deeply comprehended (Tekindal and Arsu, 2020). The main reason for selecting this design is the idea that the phenomenon of gender inequality in coaching remains invisible and implicit both at institutional level and at the level of personal experiences. The study aimed to comprehensively reveal how this phenomenon (through themes such as Invisible Labor, Sexual Objectification, Invisible Ceiling) is perceived, interpreted and experienced by coaches.

The research team consists of scholars in sports management with interests in gender sociology. Throughout the process, the researchers maintained a reflexive stance, acknowledging their own positions in the sports field to minimize bias during interviews and thematic development.

## Participant group

In selecting the study group for this research, a purposive sampling method that allows explanation of the examined phenomena and events was used. From the sub-branches of purposive sampling, criterion sampling was used to identify participants. Criterion sampling is based on the researcher including in the study those individuals who meet criteria pre-determined by a criterion list (Yildirim and Simşek, 2016).

The sample size was determined based on the principle of data saturation in qualitative research, where the depth and richness of the information provided by the 10 participants were deemed sufficient to capture the essence of the phenomenon under study.

Within this study, in order to capture the different manifestations of gender inequality in the coaching profession, it was aimed that participants meet the following main criteria:

1. Actively practicing the coaching profession.
2. Equal representation of female and male coaches (gender balance).
3. Having experience in various branches (male-dominated, female-dominated and neutral fields).

Demographic information of the participants is presented in Table 1.

**Table 1:** Demographic information of the participants.

**Participants**	**Gender**	**Age**	**Sport branch**	**Coaching certificate level**	**Years of experience**
**Participant 1**	Female	28	Swimming	2nd grade	3 Years
**Participant 2**	Female	29	Volleyball	2nd grade	5 Years
**Participant 3**	Female	33	Gymnastics	4th grade	8 Years
**Participant 4**	Female	44	Basketball	4th grade	12 Years
**Participant 5**	Female	34	Football	3rd grade	5 Years
**Participant 6**	Male	25	Wrestling	2nd grade	4 Years
**Participant 7**	Male	28	Taekwondo	3rd grade	6 Years
**Participant 8**	Male	52	Football	UEFA C	19 Years
**Participant 9**	Male	39	Basketball	4th grade	13 Years
**Participant 10**	Male	27	Fitness	3rd grade	5 Years

## Data collection

As the data collection tool in the research, a semi-structured interview form developed by the researchers was used. The interview form was prepared based on themes gender inequality, gender roles, and power relations which constitute the theoretical framework of the study. Questions were formed in light of the literature under the study's main themes:

- Invisible Labor,
- Glass Ceiling/Glass Cliff,
- Sexual Objectification.

This approach allowed participants' experiences to be examined in depth with a structured flow and to provide clarity about spontaneously emerging new situations. Interviews were recorded with participants' consent under the strict principle of confidentiality. Each interview lasted on average 45-60 min. After the interviews, the obtained data were converted into written text (transcription) in preparation for the analysis process.

## Validity and reliability

In order to ensure the rigor and credibility of the qualitative research, reliability criteria were adopted. In order to meet these criteria, the following were applied within the framework of the strategies proposed by John W. Creswell and Vicki L. Poth in 2016 (Creswell and Poth, 2016).

1. Credibility: Semi-structured in-depth interviews were used to ensure that findings accurately reflect participants' experiences; direct quotations were given at length to demonstrate the validity of themes (Invisible Labor, Sexual Objectification etc.).
2. Transferability: The sample (branch, gender, geographical location) and research procedures were explained in detail in order to increase the applicability of the study's findings to similar contexts.

## Data analysis

In the research, the Thematic Analysis method was used for in-depth analysis of qualitative data. This approach is a flexible and powerful methodology aimed at systematically identifying, analyzing and reporting patterns of meaning (themes) within data (Braun and Clarke, 2006). The analysis process began with a deductive approach because the interview questions and theoretical framework had been determined in advance. The main aim was to reveal coaches' professional experiences through hidden mechanisms of inequality identified in the literature.

The data were analyzed using thematic analysis, following a recursive six-phase process to ensure rigor and systematicity.

1. **Familiarization:** All interview recordings were transcribed verbatim. The researchers read and re-read the transcripts multiple times to gain an overall understanding of the coaches' experiences.
2. **Initial Coding:** Open coding was performed, where segments of data related to gender roles, professional boundaries, and labor were labeled.
3. **Searching for Themes**: The initial codes were grouped into broader candidate themes (e.g., challenges in female-dominated branches, interpersonal distance).
4. **Reviewing Themes**: Candidate themes were checked against the coded extracts and the entire data set to ensure they accurately reflected the participants' narratives.
5. **Defining and Naming Themes**: The final three themes Invisible Labor, Glass Ceiling & Glass Cliff, and Sexual Objectification—were defined and named to capture the essence of the phenomenon.
6. **Reporting:** The final analysis was related back to the research questions and literature, supported by direct participant quotations.

To enhance trustworthiness, two researchers independently coded a subset of the data, and discrepancies were resolved through consensus.

As a result of the analyses, three main themes explaining participants' experiences and forming the article's main sections were identified:

1. **Invisible Labor:** The additional emotional and ethical burden brought by gender.
2. **Invisible Ceiling & Glass Cliff:** Systemic barriers in career paths and the privilege/burden of risky assignments.
3. **Sexual Objectification:** How the body takes precedence over professional competence and leads to vocational constraints.

The thematic analysis process was completed by organizing the conceptual codes obtained into these three main themes. In the findings section, direct quotations from participants reflecting these themes were provided to demonstrate their validity.

Reflexivity throughout the analysis, the research team maintained a reflexive stance. As scholars in the field of sports management, we acknowledged our preconceived notions about gender dynamics in coaching. To minimize bias, we engaged in regular debriefing sessions, ensuring that the interpretations were grounded in the participants' actual words rather than our own professional assumptions.

## Findings

**Theme 1: Invisible labor**

The data obtained from participants suggest that the theme of Invisible Labor encompasses the extra mental and emotional workload coaches carry in order to maintain gender roles, manage emotional boundaries and preserve professional relationships. Under this theme the conceptual codes identified were *Identity Roles, Emotional Expression Barrier, Professional Relationships (Relational Labor), and Emotional Violence*.

(F-P1) clearly expresses the extra burden caused by the role of “emotional sensitivity” and “empathy” assigned to women in the profession, while male coach (M-P1) faces a totally opposite pressure: the obligation to remain continuously “rational” and “detached.” This creates an Invisible Labor burden under the heading of Identity Roles for both genders.

F-P1 stated that even her innate tendency is turned into a burden:

“*I'm by nature an emotional person and the expectation of emotional sensitivity and empathy from female coaches places an additional emotional burden on me. I feel that my emotionality shadows my professional seriousness*.” (F-P1)

“*The doors to advancement aren't entirely closed to me, but it feels like for my male colleagues these doors open with less questioning and less effort. This made me feel like men naturally take a more advantaged career path*.” (F-P3)

M-P1, on the other hand, described the pressure of the Emotional Expression Barrier created by the role expected of him “rational” and “detached”:

“*In order to maintain my professional seriousness in the eyes of other coaches, I've had to restrict my natural ease and everyday communication, which indirectly affects my social relations and the way I express myself*.” (M-P1)

The obligation to manage professional relationships as dictated by gender gives rise particularly for male coaches to a continuous burden of “proving ethical reliability,” creating a form of Emotional Violence.

Participant M-P4 summarized the Invisible Labor stemming from this Sense of Insecurity placed on him:

“*As a male fitness coach I feel there's a hidden sense of distrust over me…I constantly feel the need to be extra careful and distant even in social settings*.” (M-P4)

This continual demand for heightened caution and distance is the concretized form of the Emotional Violence burden experienced by male coaches in professional interactions. Female coaches, on the other hand, showed that their natural relationship of trust built with female members brought professional advantage, but that this was essentially a relationship management with no real return.

The theme of Invisible Labor revealed that coaches carry extra mental and emotional loads based on gender to sustain their professional roles. Female coaches faced risk of having their professional seriousness overshadowed due to expectations of emotional sensitivity and empathy forced by societal stereotypes, and they lived with a constant need to prove themselves. In contrast, male coaches confronted a heavy Emotional Expression Barrier because the necessity to maintain the “unshakeable leader” role and preserve ethical boundaries restricted their ability to communicate naturally. These findings show that coaches of both genders are in a state of intense, unseen effort unpaid and unrecognized imposed on professional identity.

**Theme 2: Invisible ceiling & glass cliff**

The data from participants show that this theme reveals the determining role of gender in coaches' career progression, access to leadership positions and distribution of professional responsibilities. The conceptual codes prominent in this theme were: *Authority Credit, Merit, Leadership, Role Model, Double Standard, Equality, Bias and General Acceptance*.

Findings indicated that male coaches, particularly in male-dominated branches, were recognized from the start of their careers by systemic Authority Credit; whereas female coaches had to continuously have their Merit questioned and struggled with Double Standards. This condition points to an Invisible Ceiling mechanism in which men reach leadership positions with less difficulty.

Male coaches were exempt from the burden of proving professional competence and were received with natural respect, particularly in male-dominated sports branches. Female coaches, however, were subjected to continuous questioning of their Leadership capacities even in the earliest stages of their careers. Basketball coach F-P3 stated this prejudice as follows:

“*The biggest question in the minds of management was whether I could ‘establish authority in the locker room' or whether players would see me not as a leader but just as a ‘sister*'. (F-P3)”

“*This situation split me in two: On the one hand I felt the pressure to prove my tactical knowledge and expertise more than my male colleagues I had almost no luxury to make a mistake*.” (F-P3)

Football coach M-P5 clearly expressed the systemic privilege and Authority Credit he received as he began his career:

“*Because I began my career in a branch that is mostly composed of men football I didn't encounter any prejudice or unusual expectation based on my gender… From the start I was received with natural respect and authority*.” (M-P5)

Another male coach working in the same branch described the ease he experienced compared to his female colleagues under the code General Acceptance:

“*A female coach candidate with the same education and experience is seen by management as a “risky choice,” whereas I am accepted for those positions as a natural, “reliable” one and someone from inside the community*.” (M-P3)

Despite wage equality, findings showed that Double Standards applied in workload and expectations caused inequality in career quality. Male coaches, though receiving equal pay, were often assigned to more demanding and less risk-free tasks, thus exposed to hidden inequality. Fitness coach M-P1 explained this situation as:

“*We male coaches are automatically assigned to shifts that last longer or finish later; this means handling duties that carry greater security risk and restrict our social life more, even though we earn the same salary*.” (M-P1)

On the other hand, female volleyball team coach M-P2 noted that not being entrusted with the “emotional workload” was also a Double Standard:

“*When personal or emotional problems emerged with female athletes, management tended to hand responsibility to female assistants or physiotherapists. I was expected only to lead in technical and tactical matters. This in effect meant I was exempted from the ‘emotional labour'*.” (M-P2)

Female coaches, despite having competence, faced market limitations due to their gender and were directed to tasks carrying Glass Cliff potential. Fitness/Pilates coach F-P5 summarized this as:

“*In areas perceived as more “hard” like weight training or strength development—my rate of preference, especially by male members, remained low. This created an involuntary effect that restricted my area of expertise and market, due to members' gender-based expectations of training intensity rather than my technical knowledge*.” (F-P5)

The findings of Invisible Ceiling & Glass Cliff revealed that career trajectories are shaped by gender-based systemic barriers and privileges. Male coaches, especially in male-dominated branches, were met with natural respect and Authority Credit and arrived in Leadership positions with less questioning. Female coaches, however, even when competent, struggle in a structure where their Merit is constantly questioned, they have almost no luxury to make mistakes and face Double Standards in institutional decisions. Furthermore, the findings pointed out that even when pay equality exists, men are steered to less risky and more prestigious technical duties and women are diverted to tasks constrained by emotional labor or market limits a hidden inequality of assignments.

**Theme 3: Sexual objectification**

The data from participants show that this theme reveals how coaches' professional competencies are overshadowed by sexual perceptions based on their gender and how they are restricted in the professional field due to the necessity to preserve ethical boundaries. The conceptual codes within this theme were*: Exclusion, Restriction, Communication Barrier, and Professional Competence*.

Findings show that male coaches, especially in interactions with opposite-sex members, have to be excessively cautious because of the risk of being perceived as a potential sexual threat, leading to a Communication Barrier. Female coaches, on the other hand, experienced that their body image overshadowed their *Professional Competence* and that they faced *Exclusion* in more “hard” branches.

Male coaches constantly battled a sense of distrust, particularly when interacting with female members, and this created Communication Barrier and Restriction in their professional relationships. Fitness coach E-K1 explained how he was perceived due to the combination of his gender and profession and the restrictions he experienced because of this:

“*It's as though as a male coach I have potential to be seen automatically as “not trustworthy”. This situation made me constantly feel the need to be excessively cautious and distant even in social environments. To maintain my professional seriousness in others' eyes, I've had to restrict my natural ease and communication*.” (M-P1)

Male volleyball coach M-P2 working with a female team also experienced a similar Communication Barrier and observed that the athletes were hesitant to share personal problems:

“*The most striking disadvantage I experienced working with a female team was the hesitation in establishing trust and personal closeness with players. No matter how professional and empathetic my support was, my gender created a psychological barrier between us*.” (M-P2)

Female coaches, on the other hand, reported that their Professional Competence and fields of expertise were questioned due to body image or the traditional gender of the branch. This led to their limitation and Exclusion. Gymnastics coach F-P2 stated that women are conventionally directed to “less hard” branches:

“*I experienced difficulty with parents as well; while I found it easier to communicate with female parents, the fathers of young athletes were generally more distant and critical toward me, as though they had the prejudice that I would only focus on the physical aspect of sport.”* (F-P2)

The findings associated with the theme of Sexual Objectification showed how coaches' professional identity is restricted by sexual assumptions and body stereotypes. Male coaches, particularly in interactions with opposite-sex members, carried the risk of being perceived as a potential sexual suspect, thus facing the compulsory distance and a Communication Barrier. Female coaches, meanwhile, indicated that their professional competence and fields of expertise were restricted by gender-imposed body image and expectations of toughness, and this led to their Exclusion. These findings reveal that coaches face serious restrictions in the professional environment stemming from sexual assumptions.

## Discussion

The findings of this study provide a comprehensive understanding of how gender dynamics shape the coaching profession. By adopting Bronfenbrenner's ecological systems theory, this study maps gender inequality beyond individual interactions. It situates the “Invisible Labor” at the interpersonal level, the “Glass Ceiling” at the organizational/exo-system level, and the overarching gender stereotypes and ‘Sexual Objectification' at the macro-systemic/socio-cultural level. This multi-layered perspective reveals that the challenges faced by coaches are not isolated incidents but are embedded within broader social and institutional structures.

The findings of this study regarding the barriers faced by female coaches align significantly with existing literature, particularly regarding the “Glass Ceiling” and professional competence. Consistent with Norman and French (2013). who observed that women coaches at senior levels are a minority and struggle with professional continuity, our participants emphasized that male coaches are more frequently preferred for upper-level appointments. This establishes a male-dominated profession where women's competencies are continuously questioned. This aligns with McInnis (2017) and Martin (2013), who identified that despite equal membership numbers in associations, women are under-represented in managerial positions due to the obligation to prove themselves and systemic exclusion. West et al. (2001) describe this as a strategic masculinization of the coaching profession.

Furthermore, societal gender roles significantly impact professional practice. Female participants reported being labeled as “mothers” or “sisters” rather than coaches, forcing them to adopt authoritarian behaviors to gain respect, a finding paralleled by Norman (2021). Additionally, domestic responsibilities create a conflict known as the “double burden.” (Imeson, 2017), (Walker, 2016) and (Imeson, 2017) all support our finding that household duties and the perceived unsuitability of women for late-night trainings constitute major obstacles to professional progression and promotion for female coaches.

Uniquely, our research expands the discourse by highlighting the constraints on male coaches, supporting (Connell, 2005) assertion that gender hierarchies also negatively affect men who do not conform to hegemonic norms. The finding that male coaches perceive themselves as a ‘potential threat' aligns with Norman and French (2013). who noted that male coaches maintain excessive formality to avoid allegations of impropriety. Kempe-Bergman et al. (2020) confirm that this compulsory distance prevents the formation of close, supportive coach-athlete relationships often seen with female coaches. Moreover, Elling et al. (2018) describe andro-centric structures; our study adds that these structures paradoxically limit the market share of male coaches in one-on-one consulting and personal training, where female coaches gain a competitive advantage due to being perceived as “safer.”

In light of these findings, it is essential to interpret the “dual structure” of gender inequality through a multi-layered lens. By adopting Bronfenbrenner's ecological systems theory, this study reveals that these barriers are not isolated; while the “Glass Ceiling” represents an exo-systemic (organizational) barrier, the “Invisible Labor” and communication struggles operate at the interpersonal level, all of which are governed by a macro-systemic (cultural) patriarchal ideology.

However, a critical distinction must be made to ensure conceptual clarity and avoid an unintended false equivalence. While this study identifies significant constraints for both genders, the nature of these challenges differs fundamentally. The “Glass Ceiling” and “Invisible Labour” experienced by female coaches are rooted in long-standing, systemic, and institutionalized gender hierarchies that historically marginalize women in sport leadership, reflecting a liberal feminist concern regarding structural exclusion. In contrast, the professional boundaries and communication barriers reported by male coaches—often tied to fears of being perceived as a “sexual threat”—represent contemporary ethical and professional constraints shaped by evolving social sensitivities and the pressures of hegemonic masculinity. Recognizing this distinction is vital: women's struggles reflect a structural disadvantage in career progression and power acquisition, whereas men's constraints reflect a tension between traditional coaching roles and modern professional safety norms.

## Conclusion

The main conclusion of this research is that gender inequality in the coaching profession is not limited to visible distinctions such as pay and promotion, but operates through a multi-layered, systematic problem via mechanisms such as Invisible Labor, the Glass Ceiling/Glass Cliff, and body-based Sexual Objectification. The study concludes that gender inequality possesses a dual structure: while female coaches struggle with exclusion and competence questioning, male coaches are subjected to a “Communication Barrier” and professional distancing due to the risk of being perceived as potential sexual threats.

The relevance of this study lies in its demonstration that current academic and social discourse, which largely focuses unilaterally on the disadvantaged position of women, creates a narrowing that undermines the goal of inclusive gender equality. Focusing only on women's disadvantages leads to the invisibility of the restrictions faced by men. To achieve genuine equality in sports, policies must be broadened to recognize and address the specific constraints, professional injustices, and role expectations imposed on all genders.

## Data Availability

The raw data supporting the conclusions of this article will be made available by the authors, without undue reservation.
